# Compliance and Outcome of Elderly Patients Treated in the Concurrent Once-Daily Versus Twice-Daily Radiotherapy (CONVERT) Trial

**DOI:** 10.1016/j.jtho.2018.09.027

**Published:** 2019-01

**Authors:** Marianna Christodoulou, Fiona Blackhall, Hitesh Mistry, Ahmet Leylek, Joost Knegjens, Vincent Remouchamps, Isabelle Martel-Lafay, Núria Farré, Matjaž Zwitter, Delphine Lerouge, Nicolas Pourel, Henri Janicot, Arnaud Scherpereel, Caroline Tissing-Tan, Karin Peignaux, Xavier Geets, Krzysztof Konopa, Corinne Faivre-Finn

**Affiliations:** a Department of Radiotherapy Related Research, The Christie NHS Foundation Trust, Manchester, United Kingdom; b Division of Cancer Sciences, School of Medical Sciences, Faculty of Biology, Medicine and Health, University of Manchester, Manchester, UK; c CancerCare Manitoba, Winnipeg, Canada; d The Netherlands Cancer Institute – Antoni van Leeuwenhoek Hospital, Amsterdam, Netherlands; e CHU UCL Namur, site Ste Elisabeth, Namur, Belgium; f Centre Léon Bérard, Lyon, France; g Hospital de la Santa Creu i Sant Pau, Barcelona, Spain; h Institute of Oncology, Ljubljana, Slovenia; i Centre François Baclesse, Caen, France; j Institut Sainte-Catherine, Avignon, France; k CHU Gabriel Montpied, Clermont-Ferrand, France; l Hospital of the University (CHRU) of Lille, Lille, France; m Radiotherapiegroep, Arnhem, Netherlands; n Georges Francois LECLERC Center, Dijon, France; o Cliniques universitaires Saint-Luc, MIRO-IREC-UCL, Brussels, Belgium; p Medical University of Gdansk, Gdansk, Poland

**Keywords:** SCLC, Limited stage, Radiotherapy, Elderly, Chemotherapy

## Abstract

**Introduction:**

There is a lack of data on the efficacy and safety of concurrent chemoradiotherapy in elderly, limited-stage, patients with SCLC.

**Methods:**

We compared outcomes of patients 70 years of age or older versus younger patients within the Concurrent Once-daily Versus twice-daily RadioTherapy (CONVERT) trial. Patients were randomized to receive 45 Gy/30 twice-daily fractions/19 days or 66 Gy/33 once-daily fractions/45 days concurrently with platinum-based chemotherapy. Overall survival and progression-free survival were evaluated using Kaplan-Meier methodology and Cox proportional hazards regression.

**Results:**

Of 547 patients randomized between April 2008 and November 2013, 57 did not receive protocol treatment and were excluded. Of the 490 patients included, 67 (14%) were 70 years of age or older (median age: 73 years; range: 70–82). Fewer older patients received the optimal number of radiotherapy fractions (73% versus 85%; *p* = 0.03); however, chemotherapy compliance was similar in both groups (*p* = 0.24). Neutropenia grade 3/4 occurred more frequently in the elderly (84% versus 70%; *p* = 0.02) but rates of neutropenic sepsis (4% versus 7%; *p* = 0.07) and death (3% versus 1.4%; *p* = 0.67) were similar in both groups. With a median follow-up of 46 months; median survival in the elderly versus younger groups was 29 (95% confidence interval [CI]: 21–39) versus 30 months (95% CI: 26–35), respectively; (hazard ratio: 1.15, 95% CI: 0.84–1.59; *p* = 0.38). Median time to progression in the elderly versus younger groups was 18 months (95% CI: 13–31) versus 16 months (95% CI: 14–19), respectively (hazard ratio: 1.04, 95% CI: 0.76–1.41; *p* = 0.81).

**Conclusions:**

Concurrent chemoradiotherapy with modern radiotherapy techniques should be a treatment option for fit, older patients.

## Introduction

Lung cancer is a disease of the elderly with a median age of 71 years at diagnosis.^1^ Up to 13% of all lung cancer diagnoses are SCLC and of those 30% are defined as limited-stage disease (LS-SCLC) or stage I-III according to TNM classification.^2^, ^3^, ^4^ The prognosis of LS-SCLC is poor despite optimal treatment.^2^, ^3^ The current standard of care for fit patients with LS-SCLC is thoracic radiotherapy delivered concurrently with platinum doublet chemotherapy followed by prophylactic cranial irradiation (PCI) in patients without progressive disease.^5^ The optimal radiotherapy timing and schedule was investigated in the landmark Intergroup 0096 study which showed improved survival with twice-daily concurrent chemoradiotherapy (45 Gy/30 fractions over 3 weeks) compared to once-daily treatment (45 Gy/25 fractions over 5 weeks) at the expense of higher rates of severe esophagitis.^6^ Following the Intergroup 0096 trial results in the late 1990s, concerns regarding toxicity, the low dose of radiotherapy used in the once-daily arm, and logistical issues surrounding the delivery of twice-daily radiotherapy resulted in its poor adoption in routine clinical practice.^7^ The CONVERT trial compared twice-daily to once-daily concurrent chemoradiotherapy in the era of modern conformal radiotherapy in LS-SCLC.^8^ The results showed no significant difference in survival and toxicity between the two groups establishing twice-daily radiotherapy as the standard of care in good performance status LS-SCLC. Despite no significant difference in outcomes in the twice-daily and once-daily radiotherapy arms in the CONVERT trial, survival rates in both arms were higher than previously reported.^6^, ^8^ Severe toxicity was less compared to that reported in the Intergroup 0096 trial, particularly radiation esophagitis.^6^, ^8^ The improved outcomes were thought to reflect advances in staging and supportive management of LS-SCLC in the last 2 decades, alongside the use of modern radiotherapy techniques.

The optimal treatment for older patients with LS-SCLC is not established. The prognostic role of age remains unclear with conflicting results from retrospective studies.^9^, ^10^, ^11^, ^12^, ^13^, ^14^, ^15^ Corso et al.^16^ recently showed an overall survival (OS) benefit of 6.3 months with modern chemoradiotherapy in a population-based analysis of 8637 patients aged 70 years or older with LS-SCLC. However, there is a lack of high-level evidence to guide treatment in older patients as a result of their under-representation in clinical trials.^17^, ^18^ Consequently, and also due to concerns regarding increased treatment-related toxicity, or poorer tolerance of treatment-related toxicity, older patients are less likely to receive standard of care treatment with the potential risk of under-treatment.^19^

In this analysis, we aimed to evaluate the outcomes of older patients randomized to the CONVERT trial to inform treatment decisions in older patients in the era of modern chemoradiotherapy.

## Materials and Methods

### Trial Design

Details of the trial design have been published previously.^8^ Briefly, the CONVERT trial was an international, multicenter, phase III randomized controlled trial. The main eligibility criteria were: age 18 years of age or older; Eastern Cooperative Oncology Group performance status (PS) 0-1 (on a scale of 0 to 5, with 0 indicating no symptoms and higher numbers reflecting greater disability),^20^ patients with PS 2 due to disease-related symptoms and not comorbidities; maximum of one of the following adverse biochemical factors: serum alkaline phosphatase more than 1.5 times the upper limit of normal, serum sodium less than the lower limit of normal, serum lactate dehydrogenase more than the upper limit of normal. Patients were randomized to receive either twice-daily radiotherapy (45 Gy/30 twice-daily fractions over 19 days) or once-daily radiotherapy (66 Gy/33 daily fractions over 45 days) concurrently with chemotherapy. Radiotherapy commenced on day 22 of cycle 1. Three-dimensional conformal radiotherapy was mandatory and elective nodal irradiation was not permitted. Staging with positron-emission tomography – computed tomography and the use of intensity-modulated radiotherapy were permitted but not mandated. A radiotherapy quality assurance program was set up, the details of which have been reported previously.^8^ Chemotherapy consisted of 4 to 6 cycles of cisplatin and etoposide every 3 weeks in both groups (etoposide 100 mg/m^2^ intravenously on days 1–3 and cisplatin 75 mg/m^2^ intravenously on day 1 or cisplatin 25 mg/m^2^ intravenously on days 1–3 of the cycle). Each center elected to prescribe 4 or 6 cycles for all their trial patients. Patients without evidence of progressive disease on computed tomography scan and with no clinical evidence of brain metastases were offered PCI no later than 6 weeks following the last cycle of chemotherapy. There was no upper age limit in the CONVERT trial.

Patients were followed-up for 5 years after treatment. All participants gave written informed consent to participate and the study was conducted according to the Declaration of Helsinki and Good Clinical Practice Guidelines. The protocol was approved by the institutional review board or the research ethics committee at each study center.

### Outcomes and Statistical Analysis

The outcomes of the age-subgroup analysis were OS (defined as the time from randomization until death from any cause); progression-free survival (PFS) measured from randomization to the first date of any local or metastatic disease or, if missing, date of death; acute toxicity (defined as toxicity occurring between the start and up to 3 months after completion of treatment according to the Common Terminology Criteria for Adverse Events [version 3.0])^21^; and treatment compliance. OS and PFS were estimated using the Kaplan-Meier method and compared using the log-rank test. Age groups were compared using Pearson chi-squared and Fisher’s exact tests where appropriate and continuous variables were compared using Mann-Whitney tests. The statistical analysis was performed using Stata 13.1.

## Results

### Patients

**Figure 1:**
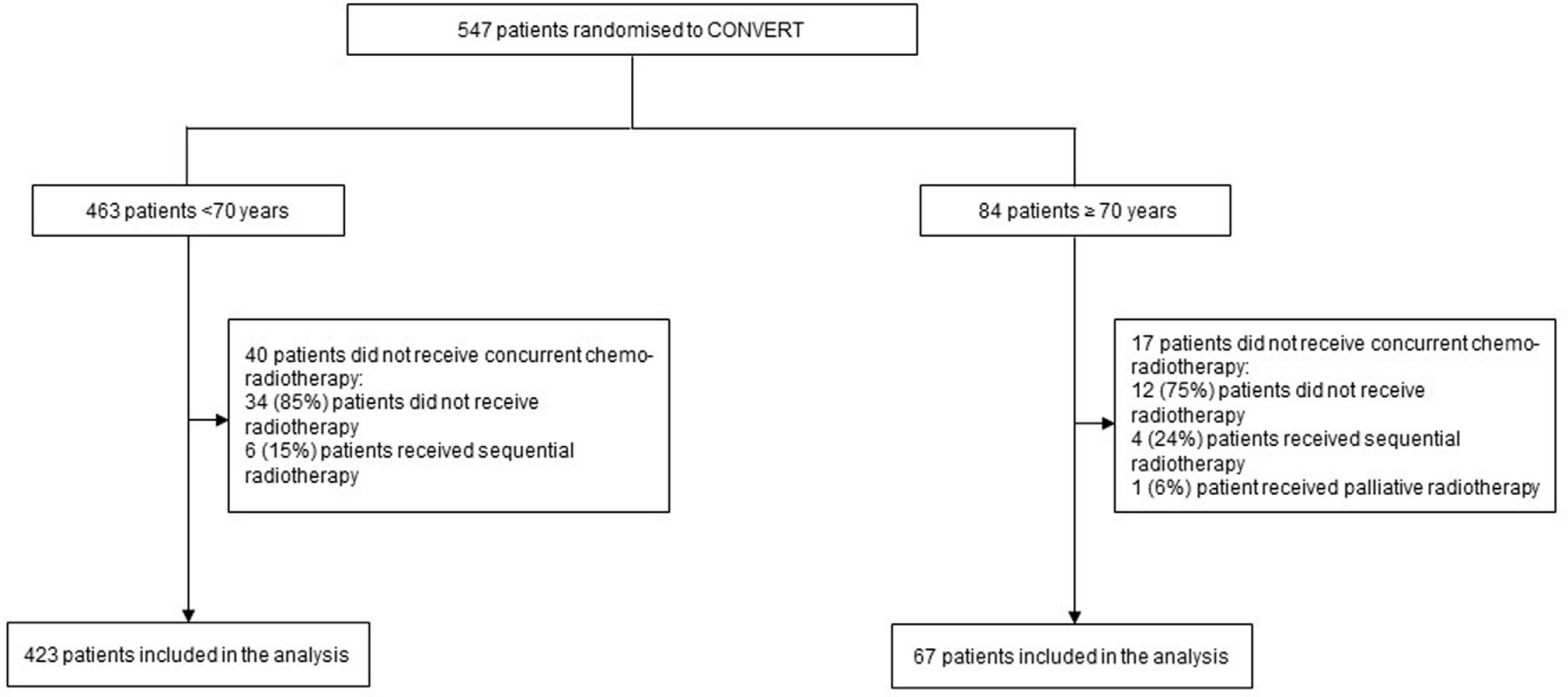
Patient allocation to age groups.

Between April 2008 and November 2013, 547 patients were randomized to the CONVERT trial. For the purposes of the age-subgroup analysis, 57 patients (32 from the once-daily and 25 from the twice-daily treatment arm, respectively) were excluded because they did not receive protocol treatment; 46 did not receive any radiotherapy, 10 received sequential radiotherapy, and 1 patient received palliative radiotherapy (Fig. 1). More older patients were excluded from this analysis compared to their younger counterparts (20% versus 9%, respectively).

Of the 490 patients included in this analysis, 67 (14%) were aged 70 years or older with a median age of 73 years (range: 70–82 years). There were 21 patients aged 75 years or older and 4 patients aged 80 years or older. The median age of the younger group was 60 years (range: 29–70 years). Among the patients 70 years or older, 29 (43%) and 38 (57%) were randomized to the twice-daily and once-daily radiotherapy arms, respectively. Baseline characteristics (Table 1) were well balanced apart from more male patients (*p* = 0.02) and ex-smokers (*p* = 0.003) in the elderly group. There were no statistically significant differences in PS or dyspnea score among the two age groups (Table 1). Notably, there were only two older patients with a PS of 2; one was randomized to the twice-daily treatment arm, and one to the once-daily arm.

**Table 1:** Patients' Characteristics at Baseline per Age Group

	Age Group		*p* Value
	<70 (%) n = 423	≥70 (%) n = 67	
Age (years)			
Median (range)	60 (29–70)	73 (70–82)	
Treatment			
BD	220 (52)	29 (43)	*0.180*
OD	203 (48)	38 (57)	
Sex			
Male	225 (53)	46 (69)	*0.020*
Female	198 (47)	21 (31)	
ECOG PS			
0	198 (47)	29 (43)	*0.840*
1	211 (50)	36 (54)	
2	14 (3)	2 (3)	
MRC dyspnea score^40^			
0	160 (38)	19 (28)	*0.259*
1	142 (34)	29 (43)	
2	56 (13)	10 (15)	
3	36 (9)	2 (3)	
4	3 (1)	1 (1)	
Weight loss in the last 6 months			
No change	269 (64)	46 (69)	*0.200*
<5%	67 (16)	12 (18)	
5%–10%	43 (10)	1 (1)	
>10%	16 (4)	4 (6)	
Not known	28 (6)	4 (6)	
UICC/AJCC stage^4^			
I-II	69 (16)	13 (20)	*0.430*
III	331 (78)	52 (78)	
Not known	23 (5)	2 (3)	
Serum alkaline phosphatase			
≥ULN × 1.5	415 (98)	65 (97)	*0.560*
>ULN × 1.5	8 (2)	2 (3)	
Serum sodium			
≥LLN	344 (81)	52 (78)	*0.470*
<LLN	79 (19)	15 (22)	
Serum LDH			
≤ULN	318 (75)	51 (76)	*0.200*
>ULN	102 (24)	14 (21)	
Not known	3 (1)	2 (3)	
PCI			
No	42 (10)	7 (11)	*0.870*
Yes	379 (90)	59 (89)	
PET-CT staging			
No	183 (43)	25 (37)	*0.600*
Yes	239 (57)	42 (63)	
Not known	1 (<1)	0 (0)	
Smoking history^a^			
Never	5 (1)	0 (0)	*0.003*
Ex-smoker	257 (61)	55 (82)	
Current	161 (38)	12 (18)	
GTV (mL)	n = 413	n = 65	
Median (range)	81.6 (0.5-635.1)	93.4 (6.3-436.4)	*0.390*

BD, twice daily; ECOG, Eastern Cooperative Oncology Group; GTV, gross tumor volume; LDH, lactate dehydrogenase; LLN, lower limit of normal; MRC, Medical Research Council; OD, once daily; PCI, prophylactic cranial irradiation; PET-CT, Positron-emission tomography–computed tomography; PS, performance status; UICC, Union for International Cancer Control; AJCC, American Joint Committee on Cancer; ULN, upper limit of normal.

a Never smokers: adults who have never smoked a cigarette or who smoked fewer than 100 cigarettes in their entire lifetime; former smokers: adults who have smoked at least 100 cigarettes in their lifetime, but say they currently do not smoke; current smokers: adults who have smoked 100 cigarettes in their lifetime and currently smoke cigarettes every day (daily) or some days (nondaily).

### Treatment Delivered

The majority of patients received 4 chemotherapy cycles (64% elderly versus 63% younger) with no significant difference in those who received less than 4 compared to 4 or more cycles in the two groups (*p* = 0.24). A similar number of older compared to younger patients received 5 or 6 chemotherapy cycles (21% versus 26%, respectively, *p* = 0.48). Radiotherapy compliance was worse in the elderly with significantly fewer patients receiving the optimal number of fractions (more than 30) as per protocol (73% elderly versus 85% younger, *p* = 0.03) (Table 2).^8^ More younger patients completed radiotherapy within the optimal treatment time defined in the protocol (19 to 21 days in the twice-daily and 45 to 47 days in the once-daily groups, respectively), but this did not reach statistical significance (41 (61%) older versus 298 (71%) younger patients, respectively; *p* = 0.153) (Table 2).^8^

**Table 2:** Radiotherapy Compliance per Age Group

Arm (N)	Dose (Gy) n (%)			No. Fractions n (%)				Overall Treatment Time (days) n (%)			
BD *(249)*	<44	44-46	>46	<28	28-29	30	>30	<19	19	20-21	>21
<70 *(220)*	1 (0.4)	216 (98)	3 (1)	10 (5)	18 (8)	191 (87)	1 (0.4)	11 (5)	143 (65)	19 (9)	47 (21)
≥70 *(29)*	0 (0)	29 (100)	0 (0)	2 (7)	5 (17)	22 (76)	0 (0)	4 (14)	15 (52)	5 (17)	5 (17)
OD *(240)*	<60	60-62	64-68	<30	30-32	33	>33	<45	45	46-47	>47
<70 *(202)*	17 (8)	16 (8)	169 (84)	13 (6)	23 (11)	165 (82)	1 (0.5)	31 (15)	98 (49)	38 (19)	35 (17)
≥70 *(38)*	5 (13)	3 (8)	30 (79)	3 (8)	8 (21)	27 (71)	0 (0)	10 (26)	16 (42)	5 (13)	7 (18)

BD, twice-daily; OD, once-daily.

In the twice-daily radiotherapy arm, 19 of 29 older patients (66%) completed chemotherapy compared to 23 of 38 older patients (61%) in the once-daily arm. A similar proportion of elderly patients received more than 30 fractions of radiotherapy as per protocol in both treatment arms: 22 (76%) and 27 patients (71%) in the twice-daily and once-daily radiotherapy arms, respectively (Table 2).

Of the 21 patients older than 75 years, 11 did not complete their planned chemotherapy and 4 did not receive the intended radiotherapy. All 4 patients who were 80 years or older completed radiotherapy; however, only 1 completed their planned chemotherapy.

A similar proportion of patients in both groups received PCI: 59 (88%) versus 377 (89%) in the elderly and younger groups, respectively, including two older patients older than 80 years.

Thirty-seven of 57 patients who were excluded from this analysis due to treatment protocol violations did not complete chemotherapy. Within the same group, only 2 of 10 patients who received sequential radiotherapy completed treatment.

### Survival

**Figure 2:**
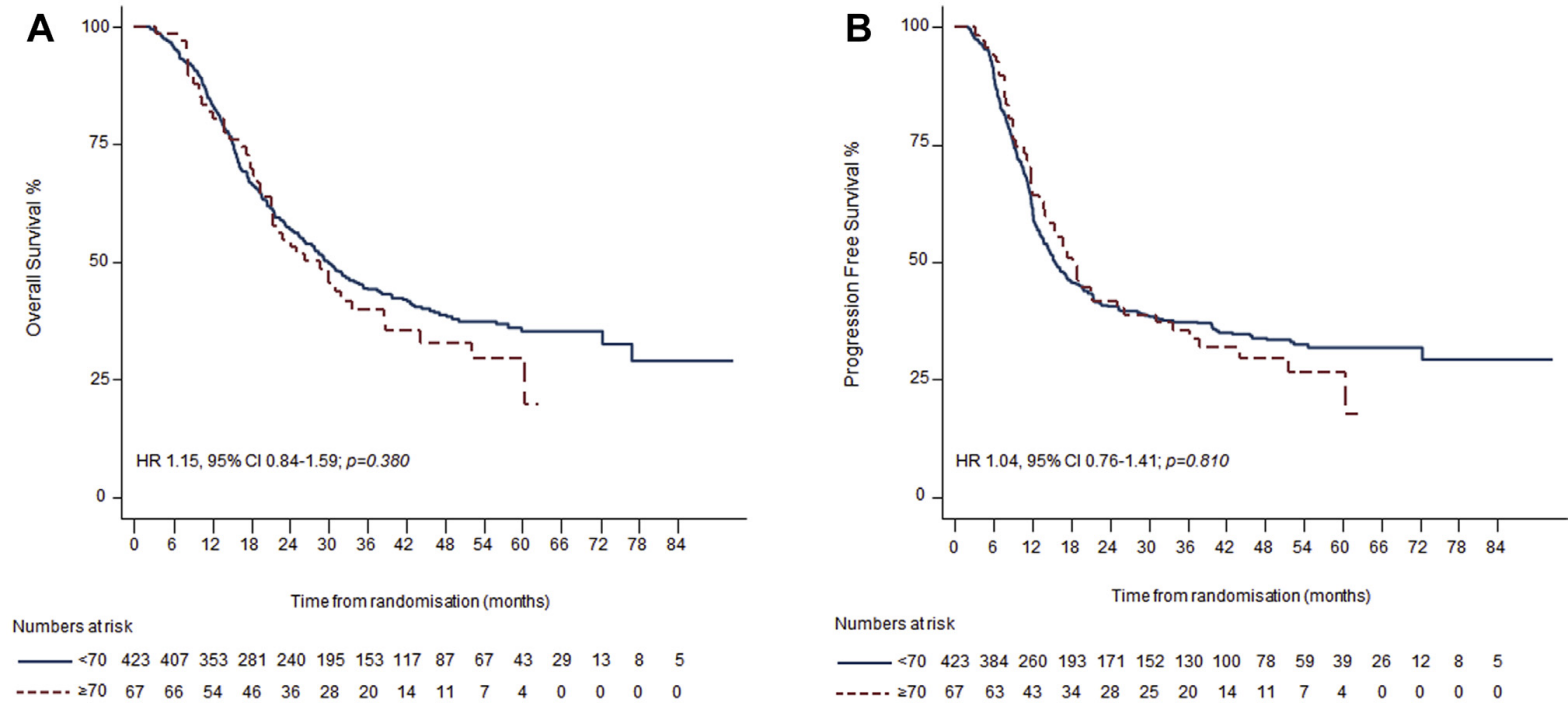
Kaplan-Meier curves for (*A*) overall survival and (*B*) progression-free survival for elderly (*dashed red line*) and younger (*blue line*) groups. Hazard ratios (HRs), 95% confidence intervals (Cis), *p* values, and number of patients at risk against yearly intervals are shown.

**Figure 3:**
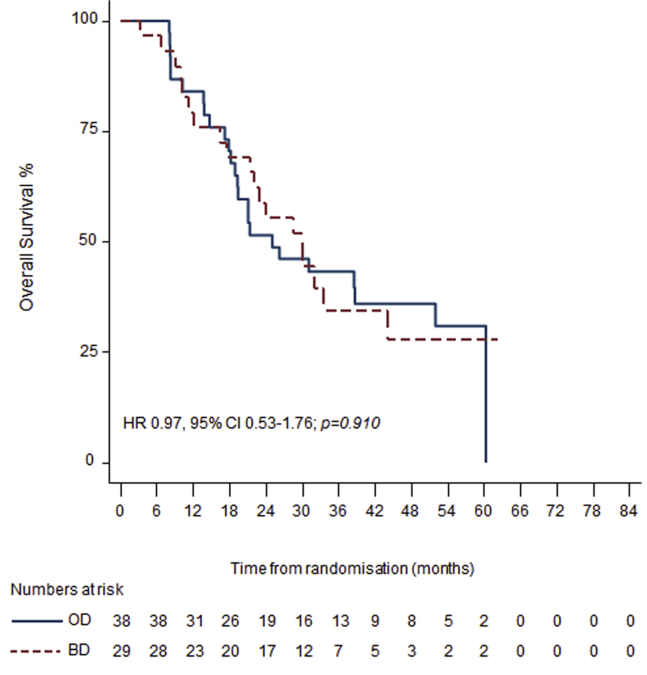
Kaplan-Meier curves for overall survival for elderly patients who received twice-daily (BD) (*dashed red line*) or once-daily (OD) radiotherapy (*blue line*). Hazard ratios (HRs), 95% confidence intervals (Cis), p values and number of patients at risk against yearly intervals are shown.

With a median follow-up of 46 months; 2-year survival was 53% (95% confidence interval [CI]: 41–64) in the elderly and 57% (95% CI: 52–61) in the younger group. Median survival was 29 months (95% CI: 21–39) in the elderly and 30 months (95% CI: 26–35) in the younger group (hazard ratio [HR] for OS: 1.15, 95% CI: 0.84–1.59; *p* = 0.38) (Fig. 2*A*). The median time to local or distal disease progression was 18 months (95% CI: 13–31) in the elderly and 16 months (95% CI: 14–19) in the younger group (HR for PFS: 1.04, 95% CI: 0.76–1.41; *p* = 0.81) (Fig. 2*B*). Two-year and OS rates were similar independent of treatment arm in elderly patients: 30 months (95% CI: 22–not reached) and 55% (95% CI: 36–71), respectively, in the twice-daily group and 26 months (95% CI: 19–not reached) and 49% (95% CI: 31–64), respectively, in the once-daily group (HR for OS: 0.97, 95% CI: 0.53–1.76; *p* = 0.91) (Fig. 3). The median OS and PFS for the protocol violation group was 11 months (95% CI: 8–17) and 7 months (95% CI: 5–12) respectively.

### Acute Toxicity

Table 3 summarizes the rates of grade 3/4 acute toxicity. Older patients suffered from significantly more neutropenia (84% versus 70%, *p* = 0.02) and thrombocytopenia (28% versus 18%, *p* = 0.05) compared to their younger counterparts. However, this did not translate into higher rates of neutropenic sepsis (4% versus 7%; *p* = 0.07), hospitalization (duration of inpatient stay in days, median [range]; 0 [0–42] versus 0 [0–50]; *p =* 0.21), and transfusion of red blood cells (43% versus 34%; *p* = 0.14) or of platelets (9% versus 6%; *p =* 0.39) in the elderly and younger groups, respectively. The rates of grade 3/4 radiation esophagitis (19% versus 20%; *p =* 0.870) and grade 3/4 pneumonitis (3% versus 2%; *p* = 0.31) were similar in the elderly and younger groups, respectively.

**Table 3:** Acute Grade 3/4 Toxicity per Age Group

Acute Grade 3/4 Toxicity	<70, n (%) N = 423	≥70, n (%) N = 67	*p* Value
Neutropenia	293 (70)	56 (84)	*0.020*
Neutropenic sepsis	29 (7)	3 (4)	*0.070*
Infection	49 (12)	9 (13)	*0.670*
Thrombocytopenia	77 (18)	19 (28)	*0.050*
Radiation pneumonitis	6 (2)	2 (3)	*0.310*
Esophagitis	85 (20)	13 (19)	*0.870*
Vomiting	22 (5)	2 (3)	*0.070*

In the group older than 75 years (n = 21), acute grade 3/4 radiation pneumonitis was reported in one patient (5%), grade 3/4 esophagitis in five patients (24%), grade 3/4 neutropenic sepsis in one patient (5%), and grade 3/4 vomiting in two patients (10%) as shown in Table 4. All four patients aged 80 years of age or older developed significant toxicity: one patient experienced grade 3 esophagitis; one patient experienced grade 4 leucopenia and grade 3 urinary infection; one patient experienced grade 4 neutropenia, grade 2 pulmonary edema, and grade 3 cardiac ischemic infarction; and one patient died from peripheral arterial ischemia, pancytopenia, and neutropenic sepsis.

**Table 4:** Acute Grade 3/4 Toxicity in Patients ≥75 Years Old

Acute G3/4 Toxicity	≥75, n (%) N = 21
Neutropenia	16 (76)
Neutropenic sepsis	1 (5)
Infection	2 (10)
Thrombocytopenia	6 (29)
Radiation pneumonitis	1 (5)
Esophagitis	5 (24)
Vomiting	2 (10)

Grade 3/4 acute toxicity in elderly patients in the twice-daily radiotherapy group (n = 29) and once-daily radiotherapy group (n = 38) was recorded as: radiation pneumonitis 0 (0%) versus 2 (5%), esophagitis 4 (14%) versus 9 (24%), neutropenic sepsis 3 (10%) versus 0 (0%), thrombocytopenia 10 (34%) versus 9 (23%), and vomiting 2 (7%) versus 0 (0%), respectively.

There were eight treatment-related deaths, two in the elderly and six in the younger group (3% versus 1.4%, *p* = 0.67). In the elderly group, one patient died from neutropenic sepsis and one from dementia attributed to PCI. In the younger group, causes of death included neutropenic sepsis in two patients, radiation pneumonitis in two patients, sepsis in one patient, and bronchial pneumonia in one patient. There were seven cardiovascular deaths in the younger group and none in the elderly group.

In the protocol violation group (n = 57), none of the patients developed grade 3/4 radiation pneumonitis or esophagitis. Twenty-one patients (37%) developed acute grade 3/4 neutropenia, five patients (9%) grade 3/4 infection, three patients (5%) grade 3/4 neutropenic sepsis, three patients (5%) grade 3/4 thrombocytopenia, and two patients (4%) grade 3/4 vomiting.

## Discussion

In this study, we report the results of the age-subgroup analysis of the CONVERT trial, the largest chemoradiotherapy randomized trial to date in LS-SCLC. The results showed comparable survival and toxicity between older and younger patients who participated in the trial. We defined older patients as those aged 70 years or more as this cutoff has been commonly used as the age limit for entry into lung cancer clinical trials.

Trial data to inform treatment decisions in this patient group are limited and more than 10 years old. A phase II trial of concurrent accelerated hyperfractionated radiotherapy (1.5 Gy/fraction twice daily to a total dose of 45 Gy) and carboplatin/etoposide chemotherapy showed a median survival of 15 months, 5-year survival of 13%, and tolerable toxicity in 72 fit patients with LS-SCLC aged 70 years or more.^22^ Analysis of the Surveillance, Epidemiology and End Results –Medicare data from 565 patients who were older than 65 years between 1992 and 2007 showed similar survival with the use of concurrent chemoradiotherapy with cisplatin/etoposide compared to carboplatin/etoposide (median survival 13.8% versus 13.7%, respectively).^23^ Apart from the CONVERT trial, the only other age-group analysis from a randomized controlled trial investigating concurrent chemoradiotherapy in LS-SCLC (Intergroup 0096) showed similar survival outcomes between age groups. However, worse hematological toxicity and treatment-related mortality was reported in patients older than the age of 70 years compared to their younger counterparts.^24^

In CONVERT, hematological toxicity was higher in the elderly but there was no increased risk of neutropenic sepsis or hospitalization. In contrast to the Intergroup 0096 trial, fatal toxicity was similar in the two age groups of the CONVERT trial, probably reflecting reduced rates of toxicity with the use of advanced radiotherapy techniques. However, the small group of patients aged 80 years or older in the trial experienced severe toxicity including one treatment-related death reported as dementia from PCI. In a meta-analysis of 2140 patients with SCLC, Pignon et al.^25^ showed improved survival outcomes with chemoradiotherapy (concurrent, sequential, or alternating) compared to chemotherapy alone in patients younger than 70 years, but this was not shown in older patients (relative risk of death: 1.07, 95% CI: 0.70–1.64). Although the benefit of PCI to OS and intracranial recurrence is well established in SCLC, data on the efficacy and safety of PCI in elderly patients with LS-SCLC are lacking.^26^, ^27^ Early studies report age (older than 60 years) as a factor associated with increased risk for acute and chronic neurotoxicity following PCI, the concurrent administration of chemotherapy with PCI, and a PCI dose greater than 30 Gy.^28^, ^29^, ^30^ A retrospective analysis of 1926 elderly (aged 70 years or older) LS-SCLC patients using the Surveillance, Epidemiology and End Results database showed that the survival benefit of PCI is maintained for patients older than 70 years, although no toxicity data were presented.^31^ A more recent analysis of a cohort of 151 patients aged 70 years or older who were treated with PCI at the MD Anderson Center showed no survival advantage in patients with tumors 5 cm or larger.^32^ There are very little data to guide the management of octogenarians with LS-SCLC. Most of the evidence comes from small retrospective series evaluating outcomes in patients with both limited- and extensive-stage disease, often treated with chemotherapy alone and with limited toxicity outcomes.^33^, ^34^, ^35^ Because of the small number of octogenarians in the CONVERT trial, we cannot draw any firm conclusions on the appropriate management of these patients; however, given the high rates of observed toxicity in this analysis, this should be further investigated in larger studies.

In this analysis, chemotherapy compliance was not different in the two age groups, even in patients who received more than 4 cycles of chemotherapy. Fewer older patients completed the optimal number of radiotherapy fractions; however, radiotherapy compliance was greater than 70% in the elderly with low rates of radiation pneumonitis and esophagitis, indicating that radiotherapy, even when administered in a twice-daily schedule, is well tolerated by older patients. In the Intergroup 0096 study, more older patients failed to complete 4 cycles of chemotherapy compared to their younger counterparts, although radiotherapy compliance was not reported. There were more older patients with a PS of 2 (10%) in the Intergroup 0096 study compared to CONVERT (3%), which could have contributed to the lower treatment compliance. In the CONVERT trial, the two patients in the elderly group with a PS of 2 (aged 76 and 73 years, respectively) completed treatment as per protocol, suggesting that treatment completion could be feasible in less fit patients, although the small number of patients prevents any meaningful conclusions. More older compared to younger patients were excluded from the analysis because they did not receive protocol treatment. This could be due to a number of reasons, including more aggressive disease and concerns regarding increased treatment-related complications in the elderly who generally have a higher comorbidity burden.

Our results are particularly relevant as robust evidence to guide treatment decisions in elderly LS-SCLC patients is lacking. The large retrospective analysis by Corso et al.^16^ is the only other recent study that investigated outcomes of older patients (aged 70 years or older) with LS-SCLC following chemoradiotherapy showing a survival benefit of chemoradiotherapy compared to chemotherapy alone (OS 15.6 months versus 9.3 months, respectively, *p* < 0.001).^16^ However, this population-based study did not report on toxicity. This analysis is associated with a number of limitations that restrict the generalizability of the results to elderly patients treated in routine clinical practice. Firstly, despite the absence of an upper age limit in the trial, only a small group of patients (14%) were older than the age of 70 years. Secondly, all patients except two in the elderly cohort had a PS of 0-1. These criteria only apply to a small proportion of elderly patients with LS-SCLC, which highlights the need for adequately powered, elderly-specific clinical trials or for population-based studies which may be more representative of the patients seen in the routine setting. Thirdly, due to the small numbers of patients who are 75 years or older and 80 years or older, preventing any meaningful statistical comparisons, we only provide summary statistics for treatment compliance and toxicity; therefore, the results should be interpreted with caution.

More recently, there has been interest in investigating physiological or functional parameters rather than chronological age in treatment decisions. However, there is still a lack of consensus on the definition of “elderly” patients. Introduction of tools such as the Comprehensive Geriatric Assessment (CGA), which provides a holistic, multidisciplinary assessment of the patient’s functional status, have been shown to predict cancer-related morbidity and mortality in the elderly.^36^ However, the CGA’s feasibility and effectiveness in clinical practice has not been yet established, limiting its widespread use. One of the few phase III randomized trials that evaluated treatment allocation in patients with NSCLC based on the CGA showed no difference in survival. However, patients experienced significantly less toxicity of any grade in the CGA arm.^37^ Disappointingly, prescreening methods for frailty that could differentiate fit patients able to receive standard of care treatment from those who would benefit from a CGA to personalize treatment have failed to show efficacy.^38^ Further research is required to evaluate the utility of these tools in clinical practice.^39^

In conclusion, concurrent chemoradiotherapy with modern radiotherapy techniques is safe and effective for fit, older patients with LS-SCLC. Certainly up to the age of 80 years chronological age as a sole factor should not be a barrier to this treatment being offered Future work should concentrate on establishing elderly-specific clinical trials incorporating functional assessment tools.
